# Advantages of using Genetically Elevated Lipoprotein(a) Levels in Predicting 5-Year Major Adverse Cardiovascular Events Relating to Coronary Artery Disease in Women

**DOI:** 10.31083/j.rcm2508308

**Published:** 2024-08-23

**Authors:** Aleksandr B. Shek, Rano B. Alieva, Alisher A. Abdullaev, Khurshid G. Fozilov, Shavkat U. Khoshimov, Guzal J. Abdullaeva, Darya V. Zakirova, Rano A. Kurbanova, Lilya E. Kan, Andrey R. Kim

**Affiliations:** ^1^Republican Specialized Scientific and Practical Medical Center for Cardiology, Ministry of Health of the Republic Uzbekistan, 100052 Tashkent, Uzbekistan; ^2^Center for Advanced Technologies, Ministry of Higher Education, Science and Innovation of the Republic Uzbekistan, 100174 Tashkent, Uzbekistan

**Keywords:** lipoprotein(a), qPCR, LPA kringle IV type 2 genetic polymorphism, CAD, MACE, sex differences

## Abstract

**Background:**

This study aimed to investigate major adverse cardiovascular events (MACE) in patients with coronary artery disease (CAD) over 5 years, in general, and depending on sex, lipoprotein(a) level, and number of kringle IV type 2 (KIV-2) repeats in the Lipoprotein(A) (*LPA*) gene.

**Methods:**

This study comprised 216 patients (120 women and 96 men) hospitalized with a diagnosis of “CAD, unstable angina IIB class”. The three-point risk of MACEs was assessed over 5 years: cardiovascular death, non-fatal myocardial infarction, and stroke. The number of KIV-2 repeats in the *LPA* gene was determined by quantitative real-time polymerase chain reaction (qPCR).

**Results:**

The relative risk of MACE in patients with elevated lipoprotein(a) (Lp(a)) was 2.0 (95% CI 1.04–3.87, *p* < 0.05) for quartile 4 (Q4) ≥48 mg/dL versus quartile 1 (Q1) ≤6 mg/dL. This was mainly attributable to an increase in men—relative risk (RR) 2.6 (95% CI 1.10–6.16, *p* < 0.05)—but not in women: RR 1.4 (95% CI 0.50–3.92). Mean lipoprotein(a) levels were inversely correlated with 42.5 and 7.5 for Q1 and Q4 KIV-2 repeat numbers, respectively. The relative risks of MACE for Q1 vs. Q4 KIV-2 repeats were as follows: 3.0 (95% CI 1.48–6.08, *p* < 0.001) for all patients; 3.0 (95% CI 1.20–6.55, *p* < 0.01) for men; 3.3 (95% CI 1.02–10.4, *p* < 0.05) for women.

**Conclusions:**

Quantifying kringle IV type 2 repeat copy number in the *LPA* gene using qPCR more accurately reflects the risk of major adverse cardiovascular events within 5 years in women with coronary artery disease.

## 1. Introduction

For a long time, lipoprotein(a) (Lp(a)) has not been as studied as low-density lipoprotein cholesterol (LDL-C), even though it contributes to a substantial share of residual cardiovascular risk [1, 2, 3]. Its ‘resurrection’ was fueled by genetic investigations supported by epidemiological, clinical, and experimental findings [4, 5, 6]. Its official inauguration in both European [7] and American guidelines [8] subsequently followed. Thus, owing to cardiological advancements, elevated lipoprotein(a) is now recognized as a global challenge for humankind, with a fifth—1.4 billion people—facing cardiovascular risk [9].

At the current stage, phase 3 studies of the antisense oligonucleotide (pelacarsen) [10] and phase 2 studies of small interfering ribonucleic acid (siRNA) (olpasiran) [11] are being completed. Simultaneously, the threshold levels for initiating treatment to mitigate cardiovascular risk and the necessary Lp(a) reduction for achieving clinically significant benefits are being determined [12]. Notably, recent analyses of the results from the EPIC-Norfolk [13] and Copenhagen General Population [14] studies have found sex differences in the impact of high lipoprotein(a) levels on increasing cardiovascular risk.

An analysis of the EPIC-Norfolk study revealed a higher association of Lp(a) levels with the risk of future coronary heart disease (CHD) in men compared to women. The risk of CHD incidence in men was statistically significant when Lp(a) levels were above the median, whereas for women, the risk of CHD incidence was statistically significant at Lp(a) levels above the 90th percentile [13]. In another large study, the Copenhagen General Population Study (CGPS), where the number of women (n = 37,545) slightly exceeded the number of men (n = 32,497), it was observed that even though Lp(a) levels increased by 27% in women over 50 years of age, this did not lead to an increased cardiovascular risk relative to the male cohort [14].

This study aimed to investigate major adverse cardiovascular events (MACE) over 5 years in patients with coronary artery disease (CAD), in general, and depending on sex, lipoprotein(a) level, and the kringle IV type 2 (KIV-2) repeat numbers in the Lipoprotein(A) (*LPA*) gene.

## 2. Materials and Methods

This study included 216 patients (120 women and 96 men) hospitalized with a diagnosis of ‘CAD, unstable angina (IIB class, Braunwald *et al*. [15], 1989)’ between 2016 and 2017. Patients with a history of myocardial infarction (MI) within the previous 3 months, type 2 diabetes requiring insulin therapy, atrial fibrillation, chronic heart failure above functional class I (New York Heart Association (NYHA)), chronic renal and hepatic insufficiency, long-term continuous use of lipid-lowering drugs, premenopausal hormone therapy, and early surgical menopause were excluded from the study. Women were considered post-menopausal if they had not experienced menstruation for at least 12 months due to natural menopause. Baseline treatment for the study group included statins, or a combination of statins and ezetimibe, to achieve target LDL-C levels. A recent meta-analysis of 24 eligible studies confirmed that hormone replacement therapy may significantly reduce Lp(a) concentrations [16] Early surgical menopause in women leads to a premature reduction in estrogen levels and an increase in Lp(a) levels.

Baseline treatment included statins or a combination of statins and ezetimibe to achieve target LDL-C levels. Over a five-year period, the three-point risk of MACEs, encompassing cardiovascular death, non-fatal myocardial infarction, and stroke, was assessed.

The following diagnostic and functional parameters were assessed in all patients to verify the CAD diagnosis and determine inclusion and exclusion criteria for the study: 12-lead electrocardiogram (ECG), echocardiography (EchoCG), ultrasound examination of the carotid arteries, 24-hour Holter monitoring, treadmill test, coronary angiography (if required), and biochemical tests.

### 2.1 Biochemical Tests

The blood lipid spectrum was evaluated by measuring the concentrations of total cholesterol (TC), triglycerides (TG), high-density lipoprotein cholesterol (HDL-C), and LDL-C. These were determined using the enzymatic colorimetric method on a Cobas c311 Roche-Hitachi automatic biochemical analyzer (Mannheim, Germany). Standardised reagents from the Roche were used (catalog numbers: cholesterol – 03039773190, HDL-C – 07528566190, LDL-C – 07005717190, TG – 20767107322).

The high-sensitivity C-reactive protein (hs-CRP) concentration was measured using a high-sensitivity latex-enhanced immunoturbidimetric method on the same Cobas c311 Roche-Hitachi automatic biochemical analyzer (Mannheim, Germany). Standardised reagent from the Roche was used (catalog number 04628918190).

The apolipoprotein A-I and B (ApoA-I and ApoB, respectively) levels were determined by a Cobas c311 Roche-Hitachi automatic biochemical analyzer (Germany) using the immunoturbidimetric method with monospecific antibodies to human ApoA-I and ApoB, respectively. Catalog numbers of the standardised reagents ApoA-I, ApoB – 03032566122, 03032574122.

The Lp(a) concentration (mg/dL) in blood serum was measured using the latex-enhanced immunoturbidimetric method on a Cobas c311 Roche-Hitachi automatic biochemical analyzer (Mannheim, Germany). Standardised reagent from the Roche were used (catalog number 05852625190). A serum Lp(a) concentration of up to 30 mg/dL was considered normal.

The proprotein convertase subtilisin/kexin type 9 (PCSK9) level was determined using the enzyme-linked immunosorbent assay (ELISA) method with the Human Proprotein Convertase 9/PCSK9 ELISA kit (MULTI SCIENCE, Hangzhou, China), following the standard procedure. Reference serum values for this kit, as established in 30 healthy volunteers of Chinese ethnicity, ranged from 77.7 to 249.0 ng/mL, with a mean value of 129.9 ng/mL.

To assess vitamin D status, calcidiol concentration in blood serum was determined using a Cobas e411 immunochemiluminescent analyzer (Roche-Hitachi, Mannheim, Germany) with Roche test systems (catalog number 05894913190). A calcidiol concentration in blood serum of 30–100 ng/mL was considered normal. Insufficiency was defined as a calcidiol concentration of 25–30 ng/mL and deficiency as less than 25 ng/mL.

Insulin concentration was determined using a Cobas e411 immunochemiluminescent analyzer (Roche-Hitachi, Mannheim, Germany) with Roche test systems (catalog number 12017547122). The method employs the ‘sandwich’ principle, where insulin from the sample forms a complex with a biotinylated monoclonal insulin-specific antibody and a monoclonal insulin-specific antibody labeled with a ruthenium complex. The serum insulin reference values were 2.6–24.9 mUl/mL.

Testosterone levels were also measured using the Cobas e411 immunochemiluminescent analyzer (Roche-Hitachi, Mannheim, Germany) with Roche test systems (catalog number 07027915190). This method is based on a competition principle: The sample incubates with a biotinylated monoclonal testosterone-specific antibody. After adding streptavidin-coated microparticles and a testosterone derivative labeled with a ruthenium complex, the resulting complex binds to the solid phase via biotin and streptavidin interaction.

Estradiol levels were measured using a Cobas e411 immunochemiluminescent analyzer (Roche-Hitachi, Mannheim, Germany) with Roche test systems (catalog number 06656021190). This method is based on the principle of competition, where the formation of immunocomplexes occurs during incubation of the sample with an estradiol-specific biotinylated antibody, and the number of these complexes depends on the analyte concentration in the sample.

### 2.2 Determination of Genotypic Frequencies

Genomic DNA was extracted from ethylenediaminetetraacetic acid (EDTA) blood samples (peripheral blood) from each participant. A commercially available DNeasy Blood kit (Qiagen, Germantown, MD, USA) was utilized following the manufacturer’s protocol. After measuring the concentration and purity photometrically, DNA aliquots were prepared with a working concentration of approximately 10 ng/µL and stored at –20 °C for further analysis.

The number of KIV-2 repeats in the *LPA* gene was determined using quantitative real-time polymerase chain reaction (qPCR), as reported by Lanktree *et al*. [17]. In brief, multiplex qPCR was conducted, which included fluorescent probes alternatively labeled for an exon in the KIV-2 domain and a control region in the human genome. The relative difference in fluorescence between the probes was assessed by the difference in polymerase chain reaction (PCR) cycle threshold (ΔCT), which is utilized to estimate the number of KIV-2 repeats. Primers and probes for exons 4 and 5 of LPA KIV-2, along with RNase P as the endogenous single-copy control gene, were designed using Primer3 software (Whitehead Institute for Biomedical Research, Cambridge, MA, USA) [18]; the sequences are provided in Table 1. Multiplex PCR was performed in a 30 µL reaction containing 30 ng of DNA. After optimization, the final concentrations of primers and probes used in the multiplex reaction were as follows: KIV2 exon 4 forward and reverse: 160 nM; KIV2 exon 4 probe: 320 nM; KIV2 exon 5 forward: 320 nM; KIV2 exon 5 reverse: 240 nM; KIV2 exon 5 probe: 480 nM; RNase P forward and reverse: 80 nM; RNase P probe: 100 nM.

**Table 1.** **Primer and probe sequences for LPA-KIV2 copy number identification**.

Target	Primer and probe sequences	
LPA-KIV2 exon4	Forward	$5^{\prime }$-GTCAGGTGGGAGTACTGCAA-$3^{\prime }$
	Reverse	$5^{\prime }$-CGACGGCAGTCCCTTCTG-$3^{\prime }$
	Probe	$5^{\prime }$-ROX-CCTGACGCAATGCTCA-BHQ2-$3^{\prime }$
LPA-KIV2 exon5	Forward	$5^{\prime }$-GCACATACTCCA CCACTGTCA-$3^{\prime }$
	Reverse	$5^{\prime }$-GCGAGTGTGGTGTCATAGATGA-$3^{\prime }$
	Probe	$5^{\prime }$-6FAM-CTTGGCAGGTTCTTCC-$3^{\prime }$
RNAse P	Forward	$5^{\prime }$-AGATTTGGACCTGCGAGCG-$3^{\prime }$
	Reverse	$5^{\prime }$-GAGCGGCTGTCTCCACAAGT-$3^{\prime }$
	Probe	$5^{\prime }$-CY5-TTCTGACCTGAAGGCTCTGCGCG-BHQ2-$3^{\prime }$

Abbreviations: LPA-KIV2 exon4 and exon5, primer and probe sequences to evaluate kringle IV type 2 repeats in exons 4 and 5 of the *LPA* gene; RNAse P, ribonuclease type P.

The quantitative PCR was performed using a QuantStudio™ 5 Real-Time PCR System (Applied Biosystems, Foster City, CA, USA) with the following cycling conditions: initial denaturation at 95 °C for 1 minute, followed by 40 cycles of denaturation at 95 °C for 5 seconds and annealing/detection at 58 °C for 15 seconds.

To ensure the accuracy of the results, all reactions were performed in triplicate. Primers and fluorescent-labeled probes were specifically designed for exonic sequences that contain no reported single nucleotide polymorphisms (SNPs). The experiment focused on probes targeted to LPA exons 4 and 5, as each exon is represented once at the genomic level in every KIV-2 repeat.

As determined by qPCR, the number of KIV-2 repeats was calculated based on the difference in cycle thresholds (CT) between the target and control probes (ΔCT). For each patient, ΔCT was calculated separately for exon 4 (ΔCT4) and exon 5 (ΔCT5). Subsequently, the average difference between ΔCT4 and ΔCT5 (ΔΔCT) was computed across all patients. Finally, the average value of ΔCT4 and ΔCT5 (ΔCT) was used to represent the relative number of KIV-2 repeats for further analysis.

### 2.3 Statistical Analysis

Statistical analysis was conducted using the SPSS version 29.0 statistic software package (Chicago, DE, USA). The normality of the distribution of continuous variables was assessed using the Kolmogorov–Smirnov test with Lilliefors correction and the Shapiro–Wilk test. Descriptive analysis results are presented as mean ± standard deviation (SD), median (Me), and interquartile range (IQR: 25th to 75th percentiles). Inter-group comparisons were performed using Student’s *t*-test for data adhering to a normal distribution. When continuous variables did not follow a normal distribution, the Mann–Whitney U-test was used for assessment even after transformation. The Kruskal–Wallis test was applied to compare differences between three or more independent groups. For paired sample comparisons, the Wilcoxon criterion was utilized. Categorical variables across different groups were compared using chi-square tests with Yates correction or Fisher’s exact test when expected cell counts were less than five. Relative risk (RR) and the 95% confidence interval (CI) were calculated using the following formula: RR = (incidence in the exposed group (Group 1))/(incidence in the control group (Group 2)). A probability value of *p* < 0.05 was considered to indicate statistical significance.

The Ethics Committee of the Republican Specialized Scientific and Practical Medical Center for Cardiology approved the study protocol.

## 3. Results

In the cohort of patients randomized to the study (Table 2), there were 96 men and 120 women. Although the women with CAD were, on average, 3.5 years older (*p* < 0.05), the comparison groups did not differ in terms of comorbidity, including hypertension (HT), body mass index (BMI), and type 2 diabetes mellitus (T2DM). Similarly, there were no significant differences in the severity of the clinical course of CAD, such as a history of myocardial infarction (MI), stroke, or revascularization.

**Table 2.** **Baseline clinical and biochemical parameters of patients and 5-year MACE**.

Parameters	All (n = 216)	I Men (n = 96)	II Women (n = 120)
Men/women (%)	96/120 (44.4/55.6%*)	96	120
Age	60.0 ± 8.7	58.1 ± 11.6	61.6 ± 10.6*
Hypertension, n (%)	128 (60.4%)	63 (65.6%)	65 (55.0%)
T2DM, n (%)	55 (26.9%)	27 (28.1%)	28 (23.3%)
History of MI, n (%)	36 (17.0%)	20 (20.8%)	16 (13.3%)
History of revascularizations (PCI, CABG), n (%)	25 (11.8%)	15 (15.6%)	10 (8.3%)
History of IS, n (%)	6 (2.8%)	4 (4.2%)	2 (1.7%)
BMI, kg/m^2^	27.3 ± 3.1	27.0 ± 3.3	27.5 ± 5.2
TC, mg/dL	206.9 ± 43.1	197.6 ± 52.6	214.3 ± 52.5*
TG, mg/dL	155 (107.3–226.5)	158.5 (103.5–228.0)	154.5 (117.5–223.5)
HDL-C, mg/dL	47.9 ± 10.1	42.6 ± 9.1	52.1 ± 14.0**
VLDL-C, mg/dL	31.0 (21.3–45.0)	32.0 (21.0–46.5)	31.0 (23.5–44.5)
LDL-C, mg/dL	120.8 ± 39.3	115.4 ± 48.2	125.1 ± 48.4
ApoA-I, mg/dL	154.4 ± 25.0	138.8 ± 13.5	167.8 ± 32.4**
ApoB, mg/dL	109.3 ± 23.8	107.7 ± 25.3	110.5 ± 26.9
ApoB/ApoA-I	0.73 ± 0.19	0.78 ± 0.18	0.68 ± 0.26**
Lp(a), mg/dL	13.0 (6.0–48.0)	12.0 (5.0–41.5)	14.0 (7.0–51.5)
Glucose, mmol/L	5.4 (5.0–6.3)	5.5 (5.0–6.6)	5.4 (5.0–6.2)
Insulin, mUl/mL	14.8 (10.7–20.9)	16.1 (10.7–23.5)	14.1 (10.8–19.5)
hsCRP, mg/L	3.5 (1.8–6.7)	2.9 (1.8–6.1)	4.0 (1.8–6.7)
PCSK9, ng/mL	205.0 (143.0–321.5)	178.0 (133.0–254.0)	238.0 (172.0–393.0)**
Vitamin D, ng/mL	20.7 (14.3–27.5)	20.9 (15.1–26.1)	20.1 (11.3–29.3)
Testosterone, ng/mL	0.4 (0.2–3.9)	4.0 (2.9–4.9)	0.2 (0.1–0.3)**
Estradiol, pg/mL	13.7 (5.0–28.9)	27.8 (18.0–40.1)	5.0 (5.0–7.6)**
МАСЕs, n (%)	51 (23.6)	27 (28.1)	24 (20.0)
			RR 0.71;
			95% CI 0.44–1.15
Cardiovascular death, n (%)	10 (4.6)	6 (6.3)	4 (3.3)
MI, n (%)	33 (15.3)	18 (18.8)	15 (12.5)
IS, n (%)	8 (3.7)	3 (3.1)	5 (4.2)

*, *p* < 0.05; **, *p* < 0.01 between groups I and II. Note: values are summarized as mean ± SD. Abbreviations: T2DM, type 2 diabetes mellitus; MI, myocardial infarction; IS, ischemic stroke; CABG, coronary artery bypass grafting; PCI, percutaneous coronary intervention; TC, total cholesterol; TG, triglycerides; HDL-C, high-density lipoprotein cholesterol; LDL-C, low-density lipoprotein cholesterol; Apo, apolipoprotein; hsCRP, high-sensitive C-reactive protein; Lp(a), lipoprotein(a); MACE, major adverse cardiovascular events; BMI, body mass index; VLDL-C, very low density cholesterol; PCSK9, proprotein convertase subtilisin/kexin type 9.

Women exhibited slightly higher total cholesterol levels (*p* < 0.05), which were associated with higher HDL-C levels (*p* < 0.05). This occurred as HDL-C levels did not significantly differ between men and women. Additionally, women had slightly higher levels of ApoA-I (*p* < 0.05) and PCSK9 (*p* < 0.01). The lower ApoB:ApoA-I ratio in women can be attributed to their higher ApoA-I levels. Of the 120 women examined, 100 (83%) were postmenopausal due to age, and 20 (17%) were premenopausal. This was reflected in their blood estradiol levels. Expectedly, testosterone levels were higher in men.

In the studied CAD patient cohort, the Lp(a) distribution was right skewed, with a median value of 13.0 mg/dL and an average of 30.2 mg/dL. The median Lp(a) level was slightly higher in women at 14.0 mg/dL (interquartile range: 7.0–51.5) compared to men at 12.0 mg/dL (interquartile range: 5.0–41.5), though this difference was not statistically significant. Additionally, the 5-year risk of MACEs was not significantly different between women and men, with a RR of 0.71 and a 95% confidence interval (CI) of 0.44–1.15.

Among all patients, the first quartile (Q1) and fourth quartile (Q4) Lp(a) values were <6 mg/dL and >48 mg/dL, respectively (refer to Table 3). It is possible that due to postmenopausal status, the Q4 for Lp(a) distribution in women was >51.5 mg/dL, while in men, it was >41.5 mg/dL. As expected, the 2^-Δ⁢Δ⁢Ct^ value, which is associated with the size of apolipoprotein(a) isoforms, was significantly lower in Q4 than in Q1 among all patients (*p* < 0.001), as well as separately among men (*p* < 0.01) and women (*p* < 0.01). In all quartiles for the Lp(a) distribution, the HDL-C and ApoA-I levels were higher, and the ApoB:ApoA-I ratio was lower in women than in men. The estradiol level in women was significantly lower in all Lp(a) quartiles, which may be attributed to postmenopausal age.

**Table 3.** **Baseline biochemical parameters of patients depending on Lp(a) quartiles**.

Lp(а), quartiles	Groups	Q1	Q2–3	Q4	*р*; Q4 vs. Q1
	All (n = 216)	<6 (n = 54)	6–48 (n = 108)	>48 (n = 54)	
	Men (n = 96)	<5 (n = 24)	5–41.5 (n = 48)	>41.5 (n = 24)	
	Women (n = 120)	<7 (n = 30)	7–51.5 (n = 60)	>51.5 (n = 30)	
TC, mg/dL	All	189.9 ± 50.4	205.8 ± 50.7	206.1 ± 55.0	NS
	Men	202.2 ± 49.8	192.3 ± 52.3	203.7 ± 56.9	NS
	Women	188.8 ± 46.1	211.6 ± 52.0	207.4 ± 49.5	NS
TG, mg/dL	All	159 (109.0–225.0)	160.5 (120.5–226.0)	130.5 (101.0–236.0)	NS
	Men	185.5 (108.0–279.5)	160.5 (103.0–228.0)	124.5 (102.0–188.5)	NS
	Women	149.5 (118.0–222.0)	159.0 (113.0–217.0)	138.5 (107.0–237.0)	NS
HDL-C, mg/dL	All	44.7 ± 11.7	47.9 ± 12.8	47.9 ± 13.8	NS
	Men	40.1 ± 9.3	42.1 ± 8.9	42.9 ± 8.7	NS
	Women	48.8 ± 11.7**	53.5 ± 14.0***	52.6 ± 15.8**	NS
VLDL-C, mg/dL	All	32.0 (22.0–45.0)	32.0 (24.0–45.5)	26.0 (20.0–47.0)	NS
	Men	37.5 (21.5–56.0)	32.0 (20.5–47.5)	25.0 (20.5–37.5)	NS
	Women	29.5 (24.0–44.0)	32.0 (23.0–43.0)	27.5 (21.0–47.0)	NS
LDL-C, mg/dL	All	114.7 ± 41.6	119.6 ± 47.9	119.2 ± 50.5	NS
	Men	110.7 ± 40.1	112.4 ± 49.8	126.1 ± 52.5	NS
	Women	115.4 ± 38.8	125.7 ± 48.9	123.4 ± 46.1	NS
ApoA-I, mg/dL	All	147.2 ± 28.8	152.3 ± 28.5	164.0 ± 34.2	NS
	Men	137.1 ± 22.8	136.4 ± 15.8	144.2 ± 20.4	NS
	Women	165.1 ± 26.9***	167.1 ± 35.9***	171.2 ± 28.9***	NS
ApoB, mg/dL	All	112.0 ± 32.3	108.6 ± 28.4	109.0 ± 31.7	NS
	Men	108.4 ± 28.4	111.7 ± 37.3	110.9 ± 28.1	NS
	Women	116.1 ± 20.3	106.2 ± 27.8	111.8 ± 29.4	NS
ApoB/Apo-AI	All	0.78 ± 0.23	0.74 ± 0.28	0.69 ± 0.27	NS
	Men	0.86 ± 0.21	0.82 ± 0.26	0.79 ± 0.20	NS
	Women	0.72 ± 0.18*	0.69 ± 0.29*	0.68 ± 0.19*	NS
2^-Δ⁢Δ⁢Ct^	All	5.45 ± 1.56	5.16 ± 1.42	4.08 ± 1.42	*р* < 0.001
	Men	5.65 ± 1.64	5.19 ± 1.40	4.27 ± 1.39	*р* < 0.01
	Women	5.33 ± 1.43	5.00 ± 1.43	4.13 ± 1.68	*р* < 0.01
Glucose, mmol/L	All	5.4 (4.9–6.3)	5.5 (5.0–6.35)	5.4 (4.9–6.1)	NS
	Men	5.4 (4.9–6.2)	5.5 (5.1–6.8)	5.4 (5.0–5.8)	NS
	Women	5.4 (5.0–6.3)	5.4 (5.0–6.0)	5.4 (5.0–6.7)	NS
Insulin, mUl/mL	All	14.9 (11.4–21.3)	14.9 (10.4–23.4)	14.5 (11.0–17.2)	NS
	Men	16.7 (12.7–21.3)	17.3 (10.6–25.5)	15.7 (10.4–21.9)	NS
	Women	13.7 (11.4–20.0)	12.7 (10.3–19.5)	14.9 (11.5–18.5)	NS
hsCRP, mg/L	All	2.5 (0.9–5.0)	4.5 (2.1–6.8)	3.3 (2.0–6.6)	NS
	Men	3.0 (1.4–5.3)	2.8 (1.4–6.4)	3.6 (2.0–6.6)	NS
	Women	2.0 (1.2–6.3)	4.7 (2.5–6.7)	3.5 (2.5–7.5)	NS
PCSK9, ng/mL	All	224.5 (156.0–343.0)	193.5 (138.5–280.5)	225.0 (148.0–361.0)	NS
	Men	178.0 (140.0–245.0)	169.0 (133.0–254.0)	219.0 (129.0–270.0)	NS
	Women	258.0 (147.0–387.0)	238.0 (177.0–330.0)	227.0 (148.0–446.0)	NS
Vitamin D, ng/mL	All	21.1 (14.4–26.4)	21.3 (15.4–30.2)	18.5 (13.2–26.8)	NS
	Men	22.7 (16.6–26.8)	21.08 (16.1–25.5)	19.1 (14.6–26.4)	NS
	Women	21.0 (11.3–25.8)	23.9 (15.2–33.2)	16.4 (11–26.8)	NS
Testosterone, ng/mL	All	2.5 (0.3–3.9)	0.3 (0.1–3.7)	0.4 (0.1–4.6)	NS
	Men	3.6 (2.6–4.4)	4.0 (3.0–4.6)	4.4 (3.0–5.0)	NS
	Women	0.3 (0.2–0.4)***	0.2 (0.04–0.2)***	0.1 (0.1–2.0)***	NS
Estradiol, pg/mL	All	15.5 (6.7–38.7)	7.0 (5.0–23.0)	19.9 (5.0–38.4)	NS
	Men	29.2 (15.5–38.7)	23.0 (17.2–35.2)	31.6 (26.7–42.8)	NS
	Women	5.0 (5.0–6.8)***	5.0 (5.0–7.0)***	5.0 (5.0–15.8)***	NS

*, *p* < 0.05; **, *p* < 0.01; ***, *p* < 0.001 (intraquartile differences between males/females). NS, statistically non-significant between 4th and 1st quartiles. TC, total cholesterol; TG, triglycerides; HDL-C, high-density lipoprotein cholesterol; LDL-C, low-density lipoprotein cholesterol; VLDL-C, very low-density lipoprotein cholesterol; Apo, apolipoprotein; hsCRP, high-sensitive C-reactive protein; Lp(a), lipoprotein(a); PCSK9, proprotein convertase subtilisin/kexin type 9.

**Fig. 1.**
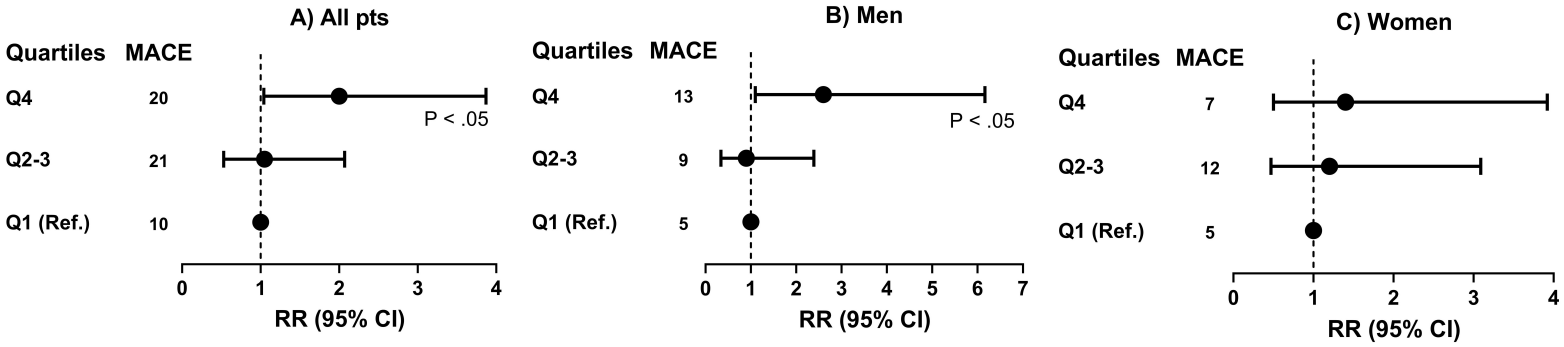
**Risk of MACEs by quartiles of lipoprotein(a) (Lp(a)) in all patients (A), and men (B)and women (C) separately**. Q1, Q2–3, Q4, quartiles of Lp(a); RR, relative risk; MACE, major adverse cardiovascular event.

The relative risk of MACEs for patients with elevated Lp(a) (Fig. 1) was 2.0 (95% CI: 1.04–3.87, *p* < 0.05) in Q4 (≥48 mg/dL) versus Q1 (≤6 mg/dL). This increase was mainly observed in men, with a RR of 2.6 (95% CI: 1.10–6.16, *p* < 0.05), but was not statistically significant in women, where the RR was 1.4 (95% CI: 0.50–3.92).

When the examined patients were divided into quartiles based on the 2^-Δ⁢Δ⁢Ct^ value (see Table 4), the lower quartile (Q1) included patients with values <3.9, and the upper quartile (Q4) >6.1. For women, these values were <3.7 and >6.1, respectively, and <4.0 and >6.3 for men, respectively. In Q1, the median Lp(a) was higher compared to Q4: 42.5 mg/dL among all patients (*p* < 0.01), 24.0 mg/dL in men (*p* < 0.01), and 46.5 mg/dL in women (*p* < 0.01). Additionally, the HDL-C and ApoA-I levels were consistently higher in all quartiles in women compared to men, while estradiol and testosterone levels were lower.

**Table 4.** **Baseline biochemical parameters of patients depending on 2^-Δ⁢Δ⁢𝐂𝐭^ quartiles**.

2^-Δ⁢Δ⁢Ct^ quartiles	Groups	Q1	Q2–3	Q4	*р*; Q1 vs. Q4
	All (n = 216)	<3.9 (n = 54)	3.9–6.1 (n = 108)	>6.1 (n = 54)	
	Men (n = 96)	<4.0 (n = 24)	4.0–6.3 (n = 48)	>6.3 (n = 24)	
	Women (n = 120)	<3.7 (n = 30)	3.7–6.1 (n = 60)	>6.1 (n = 30)	
TC, mg/dL	All	214.2 ± 50.1	205.8 ± 50.7	207.1 ± 51.1	NS
	Men	182.7 ± 53.4	203.2 ± 55.5	205.9 ± 54.9	NS
	Women	218.5 ± 48.9*	215.6 ± 52.0	215.2 ± 56.3	NS
TG, mg/dL	All	138 (102.0–183.0)	154.0 (104.3–234.5)	193.0 (133.0–245.0)	NS
	Men	133.0 (91.5–192.5)	139.5 (98.5–234.5)	201.5 (146.0–232.5)	NS
	Women	151.0 (118.0–183.0)1	153.0 (105.5–228.5)	163.5 (133.0–254.0)	NS
HDL-C, mg/dL	All	47.7 ± 15.1	47.5 ± 12.4	44.7 ± 10.6	NS
	Men	41.8 ± 8.5	44.6 ± 10.1	39.1 ± 6.5	NS
	Women	54.2 ± 16.8**	52.5 ± 13.6***	49.2 ± 11.3***	NS
VLDL-C, mg/dL	All	27.5 (20.0–37.0)	31.0 (21.0–46.8)	39.0 (27.0–50.0)	NS
	Men	26.5 (18.0–38.5)	28.0 (20.0–48.5)	41.0 (29.5–48.5)	NS
	Women	30.0 (24.0–37.0)	30.5 (21.0–45.5)	32.5 (27.0–51.0)	NS
LDL-C, mg/dL	All	128.9 ± 47.6	118.6 ± 48.7	117.0 ± 48.7	NS
	Men	105.4 ± 48.6	121.1 ± 48.1	113.3 ± 48.3	NS
	Women	131.4 ± 44.6	124.5 ± 50.0	120.0 ± 49.6	NS
ApoA-I, mg/dL	All	160.7 ± 37.0	146.8 ± 24.2	151.9 ± 28.3	NS
	Men	143.1 ± 21.3	137.3 ± 17.1	138.8 ± 19.5	NS
	Women	175.9 ± 35.3***	163.7 ± 32.1***	169.6 ± 30.1***	NS
ApoB, mg/dL	All	110.0 ± 31.2	106.8 ± 24.2	114.0 ± 31.0	NS
	Men	116.8 ± 39.1	100.3 ± 27.2	122.1 ± 34.5	NS
	Women	105.1 ± 24.4	113.4 ± 29.3*	101.9 ± 21.6*	NS
ApoB/ApoA-I	All	0.66 ± 0.26	0.74 ± 0.25	0.78 ± 0.25	NS
	Men	0.81 ± 0.27	0.74 ± 0.22	0.88 ± 0.23	NS
	Women	0.64 ± 0.24*	0.71 ± 0.28	0.63 ± 0.20***	NS
Lp(a), mg/dL	All	42.50 (10.0–80.0)	13 (9–21.5)	7.5 (5.0–20.0)	*p * < 0.01
	Men	24.0 (6–60.5)	12.74 (6–52)	5 (3.5–19.0)	*p * < 0.01
	Women	46.5 (10–80)	14 (7.5–45.5)	9 (6–20)	*p * < 0.01
Glucose, mmol/L	All	5.4 (5.0–6.0)	5.4 (4.9–6.4)	5.5 (5.2–6.7)	NS
	Men	5.6 (5.0–7.4)	5.3 (4.9–6.1)	5.6 (5.4–6.8)	NS
	Women	5.4 (5.0–5.9)	5.2 (4.8–6.2)	5.5 (5.1–6.7)	NS
Insulin, mUl/mL	All	14.5 (10.6–20.1)	14.5 (10.2–19.6)	16.0 (12.5–24.0)	NS
	Men	15.2 (9.8–23.4)	15.4 (10.6–23.8)	17.4 (14.0–23.6)	NS
	Women	15.3 (11.8–23.4)	13.5 (10.0–16.7)	13.2 (11.5–24.3)	NS
hsCRP, mg/L	All	3.9 (2.0–7.2)	3.5 (1.8–6.6)	3.5 (1.4–6.9)	NS
	Men	2.9 (2.1–6.1)	2.8 (1.4–5.2)	3.2 (0.8–8.2)	NS
	Women	4.2 (2.0–8.2)	4.6 (1.8–6.6)	3.4 (1.5–6.1)	NS
PCSK9, ng/mL	All	243.0 (161.0–330.0)	190.5 (137.3–299.5)	194.3 (136.0–313.0)	NS
	Men	229.0 (178.0–372.0)	153.0 (121.0–254.0)	163.0 (127.0–216.5)	NS
	Women	258.0 (160.0–282.0)	227.0 (173.0–446.0)*	229.5 (159.0–426.5)	NS
Vitamin D, ng/mL	All	20.9 (12.5–27.3)	21.3 (15.3–30.2)	21.5 (16.4–28.7)	NS
	Men	24.0 (17.9–27.0)	18.5 (14.4–25.5)	23.9 (18.5–26.8)	NS
	Women	17.7 (11.0–27.9)	20.6 (11.3–29.7)	20.7 (16.0–29.0)	NS
Testosterone, ng/mL	All	0.3 (0.2–4.1)	1.9 (0.1–4.0)	0.7 (0.2–3.9)	NS
	Men	4.7 (3.7–5.0)	4.0 (2.8–5.4)	3.9 (2.6–4.1)	NS
	Women	0.2 (0.0–0.23)***	0.2 (0.1–0.2)***	0.2 (0.1–0.3)***	NS
Estradiol, pg/mL	All	12.5 (5.0–29.2)	16.4 (5.0–28.1)	13.8 (5.0–39.1)	NS
	Men	28.9 (15.4–39.7)	26.7 (18.0–35.2)	39.1 (20.4–46.8)	NS
	Women	5.0 (5.0–10.1)***	5.0 (5.0–7.6)***	5.0 (5.0–9.6)***	NS

*, *p* < 0.05; **, *p* < 0.01; ***, *p* < 0.001 (intraquartile differences between males/females). NS, statistically non-significant between 4th and 1st quartiles. TC, total cholesterol; TG, triglycerides; HDL-C, high-density lipoprotein cholesterol; LDL-C, low-density lipoprotein cholesterol; VLDL-C, very low-density lipoprotein cholesterol; Apo, apolipoprotein; hsCRP, high-sensitive C-reactive protein; Lp(a), lipoprotein(a); PCSK9, proprotein convertase subtilisin/kexin type 9.

**Fig. 2.**
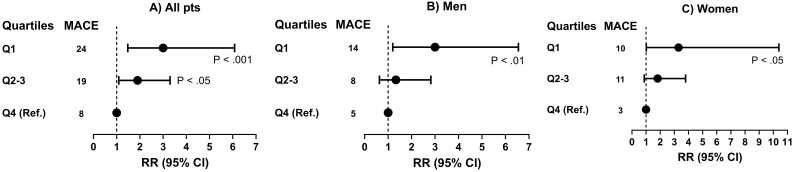
Risk of MACEs by quartiles for 2^-Δ⁢Δ⁢𝐂𝐭^* (number of KIV-2 repeats) in all patients (A) and men (B) and women (C) separately. Q1, Q2–3, Q4, quartiles of 2^-Δ⁢Δ⁢Ct^* from quantitative real-time polymerase chain reaction (qPCR), as a genetic score for lipoprotein(a) concentration, associated with apolipoprotein(a) isoform size and *LPA* KIV-2 number of repeats. RR, relative risk; MACE, major adverse cardiovascular event; KIV-2, kringle IV type 2.

The RRs of MACEs for Q1 versus Q4 according to 2^-Δ⁢Δ⁢Ct^ (number of KIV-2 repeats) were as follows: 3.0 (95% CI: 1.48–6.08, *p* < 0.001) for all patients; 3.0 (95% CI: 1.20–6.55, *p* < 0.01) for men; 3.3 (95% CI: 1.02–10.4, *p* < 0.05) for women (see Fig. 2).

## 4. Discussion

CAD is a leading cause of death in both men and women across developed and developing countries [19]. An analysis of the Women’s Health Study cohort involving female healthcare professionals revealed that in women under 55 years of age, premature CAD was more closely associated with diabetes mellitus, metabolic syndrome, hypertension, and smoking than LDL-C, non-HDL-C, and ApoB [20].

The estrogen level in women fluctuates cyclically at different periods of their lifetime, and this causes changes in the levels of lipids [21]. Additionally, decreased estradiol levels adversely affect postmenopausal lipid profiles, leading to a complex of pathogenetic issues, including endothelial dysfunction and inflammation, which in turn increase the risk of atherosclerotic cardiovascular disease (ASCVD) in women [22]. Population studies have shown that Lp(a) levels increase by 8–13% in postmenopausal women relative to premenopausal controls [12, 23, 24]. Lp(a) plasma levels are determined by the apolipoprotein(a) (apo(a)) levels, the expression of which is also modulated by estrogens [24]. The results of a recent meta-analysis [16] of 24 eligible studies confirmed that hormone replacement therapy (HRT) may significantly reduce Lp(a) concentrations compared with placebo or no treatment. In this case, oral estrogen causes a greater reduction in Lp(a) concentrations than transdermal estrogen.

Lp(a) has been recognized as an independent risk factor for cardiovascular morbidity and mortality. Recent findings from authoritative studies, including the Women’s Health Study (WHS), EPIC-Norfolk, and the CGPS—amounting to over 100,000 observations [13, 14, 25]—have highlighted sex-specific variations in its impact on cardiovascular risk.

Initially, results from three cohort studies [25] in women (WHS, n = 24,558; Women’s Health Initiative (WHI), n = 1815; Justification for the use of statins in prevention (JUPITER), n = 2569) demonstrated its limited effect on cardiovascular risk compared to the JUPITER study in men (n = 5161). Unlike men, elevated Lp(a) did not independently affect the development of endpoints in women but only in combination with an increased level of total cholesterol (>220 mg/dL).

Then, the results of the EPIC-Norfolk study [13], which investigated the association between Lp(a) levels and long-term CAD, IS, and calcific aortic valve stenosis (CAVS) in a population-based study of 25,663 men and women aged between 45 and 79 years residing in Norfolk (UK), demonstrated a link between high Lp(a) with prevalent CAD and CAVS in both men and women but not with ischemic stroke in women.

Finally, the CGPS with 37,545 women and 32,497 men aged 20–89 years, with a median age of 60 years (from 2003 to 2006 until the studied endpoint, death, or December 2018), showed that Lp(a) levels increased modestly to around age 50 in women (+27%); however, the risk of morbidity and mortality for high Lp(a) was similar in women and men above 50 [14].

The CGPS [14], which is the largest study in this context, found that Lp(a) levels increased with age in women due to decreased estradiol concentrations and estimated glomerular filtration rate (eGFR). However, despite higher levels of lipoprotein(a) in women relative to men, there was a similar risk for myocardial infarction, ischemic heart disease, ischemic stroke, aortic valve stenosis, heart failure, cardiovascular mortality, and all-cause mortality in women and men with lipoprotein(a) levels >40 mg/dL (83 nmol/L), overall and stratified for above and below age 50.

The authors believe that the modest increase in lipoprotein(a) levels selectively in women aged about 50 years observed in the CGPS challenges the current view that only one measurement of lipoprotein(a) in women and men is sufficient to determine the concentration of lipoprotein(a) over a lifetime [14]. This is especially important for women who had lipoprotein(a) measurements taken before age 50, where additional lipoprotein(a) measurements should be considered at a later stage. However, it has yet to be determined exactly how many measurements will be needed from the onset of post-menopause in women since it varies across the lifetime.

The kringle IV (KIV) repeat polymorphism explains approximately 30–70% of the variability [12] in Lp(a) concentration and allows the identification of patients with small apolipoprotein(a) isoforms. Most large-scale studies have demonstrated that apo(a) size is genetically determined and that predominantly small isoforms are associated with an increased cardiovascular risk [4, 26, 27]. In this regard, qPCR is a faster, more sensitive, and more reliable assay to detect the number of KIV2 repeats in LPA, which will facilitate risk stratification and decision-making on the initiation of therapy in women with difficult cases [28].

In our study, 100 out of 120 examined women (83%) were observed to be postmenopausal due to age. Furthermore, in the fourth quartile (Q4) of the Lp(a) distribution among women, the Lp(a) value exceeded 51.5 mg/dL, compared to >41.5 mg/dL in men (+19.4%, NS), but this did not correspond to an increased 5-year risk of MACEs in women. However, in the first quartile (Q1) of the 2^-Δ⁢Δ⁢Ct^ distribution in all patients and men and women separately, which included individuals with genetically determined pathogenic “small” apo(a) isoforms, the Lp(a) values were significantly higher than in Q4 for all cases (*p* < 0.01). In our study, this combination of elevated Lp(a) and genetic risk factors increased MACEs in both men and women equally.

## 5. Conclusions

The quantification of kringle IV type 2 repeat copy number in the *LPA* gene through qPCR more accurately reflects the risk of MACE within 5 years in women with coronary artery disease. This sex-specific observation underscores the importance of personalized medical approaches in the management and risk assessment of CAD. Alternatively, this may necessitate, in some cases, the use of inexpensive and technically accessible genetic screening methods, such as qPCR, to tailor treatment more effectively.

### Study Limitation

The present study has some limitations. It includes a relatively small number of patients with CAD (n = 216), 96 men and 120 women. Thus, further research is needed to explore the underlying mechanisms of this gender disparity and to evaluate the potential for integrating LPA gene analysis into clinical practice to improve the prediction of MACEs in CAD patients.

## Data Availability

Additional resources can be obtained from the corresponding author [Aleksandr Shek], with the consent of all authors of the article.
